# Global Public Perceptions of Genomic Data Sharing: What Shapes the Willingness to Donate DNA and Health Data?

**DOI:** 10.1016/j.ajhg.2020.08.023

**Published:** 2020-09-17

**Authors:** Anna Middleton, Richard Milne, Mohamed A. Almarri, Shamim Anwer, Jerome Atutornu, Elena E. Baranova, Paul Bevan, Maria Cerezo, Yali Cong, Christine Critchley, Josepine Fernow, Peter Goodhand, Qurratulain Hasan, Aiko Hibino, Gry Houeland, Heidi C. Howard, S. Zakir Hussain, Charlotta Ingvoldstad Malmgren, Vera L. Izhevskaya, Aleksandra Jędrzejak, Cao Jinhong, Megumi Kimura, Erika Kleiderman, Brandi Leach, Keying Liu, Deborah Mascalzoni, Álvaro Mendes, Jusaku Minari, Nan Wang, Dianne Nicol, Emilia Niemiec, Christine Patch, Jack Pollard, Barbara Prainsack, Marie Rivière, Lauren Robarts, Jonathan Roberts, Virginia Romano, Haytham A. Sheerah, James Smith, Alexandra Soulier, Claire Steed, Vigdís Stefànsdóttir, Cornelia Tandre, Adrian Thorogood, Torsten H. Voigt, Anne V. West, Go Yoshizawa, Katherine I. Morley

**Affiliations:** 1 Society and Ethics Research Group, Connecting Science, Wellcome Genome Campus, Cambridge CB10 1SA, UK; 2 Faculty of Education, University of Cambridge, Cambridge CB2 8PQ, UK; 3 Institute of Public Health, University of Cambridge, Cambridge CB2 0SR, UK; 4 Wellcome Sanger Institute, Cambridge CB10 1SA, UK; 5 Keynote IAS, New Delhi 110060, India; 6 Russian Medical Academy of Continuous Professional Education, Moscow 119049, Russia; 7 EMBL-EBI, Wellcome Genome Campus, Cambridge CB10 1SA, UK; 8 Medical Ethics Program, Peking University Health Science Center, Beijing 100191, China; 9 Department of Psychological Sciences, Swinburne University of Technology, Melbourne, VIC 3122, Australia; 10 Centre for Law and Genetics, University of Tasmania, Hobart, TAS 7001, Australia; 11 Centre for Ethics & Bioethics, Uppsala University, Uppsala SE-751 22, Sweden; 12 Ontario Institute for Cancer Research, MaRS Centre, Toronto, ON M5G 0A3, Canada; 13 Department of Genetics & Molecular Medicine, Kamineni Hospitals, Hyderabad 500 068, India; 14 SAAZ Genetics, Hyderabad 500033, India; 15 Faculty of Humanities and Social Sciences, Hirosaki University, Hirosaki 036-8560, Japan; 16 Department of Public Health and Caring Science, Uppsala University, Uppsala 751 22, Sweden; 17 Department of Molecular Medicine and Surgery, Karolinska Institutet, Solna 171 76, Sweden; 18 Research Centre for Medical Genetics, Moscow 115522, Russia; 19 Independent Scholar, Warsaw, Poland; 20 Department of Epidemiology and Biostatistics, School of Health Sciences, Wuhan University, Wuhan 430071, China; 21 Institute of Innovation Research, Hitotsubashi University, Tokyo 186-8603, Japan; 22 Centre of Genomics and Policy, McGill University, Montreal, QC H3A 0G1, Canada; 23 RAND Europe, Cambridge CB4 1YG, UK; 24 Public Health, Department of Social Medicine, Osaka University Graduate School of Medicine, Osaka 565-0871, Japan; 25 School of Public Health, Peking University Health Science Center, Beijing 100191, China; 26 EURAC, Institute of Biomedicine, Bolzano 39100, Italy; 27 UnIGENe and CGPP (Centre for Predictive and Preventive Genetics), IBMC (Institute for Molecular and Cell Biology), i3S (Instituto de Investigação e Inovação em Saúde), Universidade do Porto, Porto 4200-135, Portugal; 28 Uehiro Research Division for iPS Cell Ethics, Center for iPS Cell Research and Application (CiRA), Kyoto University, Kyoto 606-8507, Japan; 29 Genomics England, Queen Mary University of London, London EC1M 6BQ, UK; 30 Department of Political Science, University of Vienna, Vienna 1010, Austria; 31 Department of Global Health & Social Medicine, King’s College London, London WC2R 2LS, UK; 32 Diltec, Sorbonne Nouvelle, Paris 75005, France; 33 Landspitali, the National University Hospital of Iceland, Reykjavík 101, Iceland; 34 Institute of Sociology, RWTH Aachen University, Aachen 52062, Germany; 35 Indiana University Maurer School of Law, Bloomington, IN 47405, USA; 36 Work Research Institute (AFI), Oslo Metropolitan University, Oslo 0130, Norway; 37 Institute of Psychiatry, Psychology & Neuroscience, King’s College London, London SE5 8AF, UK; 38 Centre for Epidemiology and Biostatistics, Melbourne School of Global and Population Health, The University of Melbourne, Melbourne, VIC 3010, Australia; 39 Medical Ethics, Lund Universitet, Lund SE-221 00, Sweden

**Keywords:** genomic data, health data, attitudes, public, global, trust, data sharing, genomic data sharing, data donation, survey

## Abstract

Analyzing genomic data across populations is central to understanding the role of genetic factors in health and disease. Successful data sharing relies on public support, which requires attention to whether people around the world are willing to donate their data that are then subsequently shared with others for research. However, studies of such public perceptions are geographically limited and do not enable comparison. This paper presents results from a very large public survey on attitudes toward genomic data sharing. Data from 36,268 individuals across 22 countries (gathered in 15 languages) are presented. In general, publics across the world do not appear to be aware of, nor familiar with, the concepts of DNA, genetics, and genomics. Willingness to donate one’s DNA and health data for research is relatively low, and trust in the process of data’s being shared with multiple users (e.g., doctors, researchers, governments) is also low. Participants were most willing to donate DNA or health information for research when the recipient was specified as a medical doctor and least willing to donate when the recipient was a for-profit researcher. Those who were familiar with genetics and who were trusting of the users asking for data were more likely to be willing to donate. However, less than half of participants trusted more than one potential user of data, although this varied across countries. Genetic information was not uniformly seen as different from other forms of health information, but there was an association between seeing genetic information as special in some way compared to other health data and increased willingness to donate. The global perspective provided by our “Your DNA, Your Say” study is valuable for informing the development of international policy and practice for sharing genomic data. It highlights that the research community not only needs to be worthy of trust by the public, but also urgent steps need to be taken to authentically communicate why genomic research is necessary and how data donation, and subsequent sharing, is integral to this.

## Introduction

Analyzing genomic and health data across populations is central to understanding the involvement of genetic factors in health and disease.^1^^,^^2^ Responsible data sharing supported by trustworthy data governance can support the equitable delivery of genomic medicine and the right of everyone to benefit from scientific research.^3^^,^^4^

The success of data sharing relies on public support and trust.^5^ Collecting and sharing data brings to the forefront issues of privacy and questions about exploitation and uneven global distributions of scientific resources, both past and present.^6^ Data sharing should therefore be accompanied by an understanding of how members of the public, as donors of data, see and support the process of data sharing.^1^ For example, there is public hesitancy about connections between public and for-profit sectors in genomic and health research.^7^, ^8^, ^9^, ^10^, ^11^ However, research into public perceptions of genomics and biomedical data is dominated by studies from Europe and North America and rarely enables comparative study.^6^^,^^7^^,^^12^^,^^13^

This paper presents findings from a study of public perspectives on genomic data sharing, drawing on responses from 36,268 individuals across 22 countries and gathered in 15 languages. The objective of the study is to explore global public attitudes toward willingness to donate one’s DNA and health information to be shared for research (both non-profit and for-profit), together with an understanding of the factors that shape this. The study offers insight for policy makers, genomic researchers, clinicians, and governments who are implementing genomic research strategies across the world. First, we examine how the willingness to donate DNA and health information differs depending on with whom data are shared, comparing the willingness to donate to medical, non-profit, and for-profit groups across countries. Second, we examine how this willingness to donate is shaped by several factors. One factor is one’s familiarity with DNA, genetics, and genomics—either through popular culture, media, education, personal experience due to being a genetics patient or having a family history of disease, or working as a genetic health professional or genetic scientist. We grouped “DNA, genetics, and genomics” into one concept in the question about familiarity in our survey so that participants could see that the terms were linked, even if they had only heard of one of them, and we summarize this in the Results as “familiarity with genetics.” A second factor is one’s beliefs about genomic data’s being different from other health data (*cf.* “genetic exceptionalism”). A third factor is one’s trust in organizations involved in the collection, sharing, and use of data.^14^, ^15^, ^16^

In this paper we use the term “genomics” generically as a descriptor of any level of DNA testing/analysis/interpretation. In the survey itself, we used the term “DNA information” instead of “genomic data” (a term more commonly used in scientific circles) because our pilot work showed “DNA” was better understood than “genetics” or “genomics.”

## Material and Methods

### Sample

Via the international network of researchers within the Global Alliance for Genomics and Health (GA4GH), the research team invited social science, genetic counselling, and policy collaborators around the world to participate in the “Your DNA, Your Say” project through either supporting recruitment into the project or translating the survey into their native language. The “convenience” mix of countries involved in the final dataset thus reflects the reach of the GA4GH network and enthusiasm to participate in the research; all collaborators are listed as co-authors, and each will explore, in time, their own individual country data in more depth in future publications (see Video S1 for details about the project and translations). Data were collected via a cross-sectional online survey with participants recruited via the market research company Dynata. We aimed to recruit a sample that was as representative as possible of each country’s population with regard to gender, age, and education level. To this end, participant characteristics were monitored during recruitment to proactively ascertain individuals from under-represented population subgroups. Sociodemographic characteristics of participants from each country are shown in Table S1.

Video S1 Filmed Co-authors Discussing the Project and Translations

In Japan, participants were recruited through a survey research company (Cross Marketing) via the same approach. In Pakistan and India, recruitment was conducted by market research companies (Foresight and Maction, respectively) and methods were varied to account for lower internet access. In Pakistan, participants completed the questionnaire on a tablet at a central location. In India, participants completed the questionnaire on tablets provided by field researchers. Completed surveys were gathered from Argentina, Australia, Belgium, Brazil, Canada, China, Egypt, France, Germany, India, Italy, Japan, Mexico, Pakistan, Poland, Portugal, Russia, Spain, Sweden, Switzerland, the United Kingdom, and the United States of America. Participants were paid a small financial reward (<£1) for participating, and because of the nature of recruitment, there are no details on non-response rate. The study methodology, design, recruitment strategy, and process of data collection are described separately.^17^

### Measures

Our online survey contains 29 questions; background information about the landscape of genomic research and data sharing is provided via nine films that sit within the survey (see Video S2 for one of the films with Japanese subtitles), and no prior knowledge about genomics is required to participate. Details on the survey, data cleaning, and derivation of “familiarity with genetics” and “genetic exceptionalism” used in the analyses are provided in the Supplemental Material and Methods.

Video S2 One of the Nine Films in the Survey to Explain Genomic Data Sharing, Includes Japanese Subtitles

#### Trust

Participants were asked to indicate if they would trust the following people or institutions with their DNA or health information: “my medical doctor,” “any medical doctor in my country,” “any researcher at a university in my country,” “any researcher in a company in my country,” and “the government of my country.”

Response options were “I would generally trust,” “I’m just not sure,” and “I would not generally trust.” Variables were combined to create a single binary indicator of whether people reported trusting at least two of these people or organizations. Prior analysis has found that many people trust their own doctor but are less trusting of others.^16^ This binary variable aimed to capture trust across users of health and genomic data.

### Analysis

#### Sample Description

Sample characteristics were summarized with standard descriptive statistics. Bivariate relationships were evaluated with χ^2^ tests because all variables were categorical. The importance of p values was considered in the context of multiple testing.

#### Meta-Analysis

We used meta-analysis to investigate relationships between different sets of predictors and outcomes. This approach provides an estimate of the association between variables for the whole sample while also allowing exploration of between-country variation. For our outcomes, we used four measures of willingness to donate DNA and medical information related to recipient: (a) medical doctors, (b) non-profit researchers, (c) for-profit researchers, and (d) more than one of these recipients. We examined associations with three predictors (Table S3): (a) genetics familiarity, (b) genetic exceptionalism, and (c) trust in the organizations receiving the data. Analyses were adjusted for sociodemographic variables (age, gender, having children, tertiary education, and religiosity). For meta-analysis purposes, the three-category willingness to donate variables were split into two binary contrasts: unsure versus unwilling to donate and unsure versus willing to donate. This enabled us to compare participants who had a strong position with those who were unsure.

We conducted a “two-step” individual participant data meta-analysis to examine the relationship between the predictors and outcomes.^18^^,^^19^ We used a random-effects model in all analyses because we anticipated between-country differences; we estimated Cochrane’s Q and I^2^ values to test for the presence of heterogeneity. Because of the nature of the data collection approach, missing data were very limited (<5% for all questions), and therefore, complete case analyses were conducted. Results were tabulated and displayed with forest plots. All tests were two-tailed. We present and interpret p values as measures of the strength of evidence for an association rather than simply applying a threshold for statistical significance.^20^ Analyses were conducted in the R statistical software with the *meta* and *metafor* packages.^21^, ^22^, ^23^ Further details are provided in the Supplemental Material and Methods.

#### Ethics Approval

The online project is physically based at the Wellcome Genome Campus, and all data are collected and stored in encrypted files at the Wellcome Sanger Institute in Cambridge. As part of the conditions of research delivery at this research institution, the project passed ethical review by the legal counsel as well as the Human Materials and Data Management Committee to ensure that it was compliant with appropriate ethical and legal standards for participant involvement, data collection, and storage. Because the online survey is fully anonymous (and even IP addresses are not stored or shared with the research team), participants are informed that their consent is given when they choose to click off the landing page and start answering the questions. On the landing page, the purpose of the project is explained as well as what participation involves, and participants have a choice at any stage within the survey to stop answering the questions and withdraw. This ethics approval was sufficient to cover recruitment into the online survey for most of the collaborators attached to the project. The exception was Australia, where the University of Tasmania required an additional local REC process to be completed plus the addition of their own separate consent form onto the landing page of the survey for Australian participants only.

## Results

### Sample Characteristics

After data cleaning, the analysis sample included 36,268 participants from 22 countries: Argentina (919), Australia (1,212), Belgium (544), Brazil (1,349), Canada (2,966), China (3,008), Egypt (1,427), France (790), Germany (1,193), India (482), Italy (1,229), Japan (4,748), Mexico (1,347), Pakistan (925), Poland (2,904), Portugal (2,224), Russia (1,075), Spain (1,272), Sweden (821), Switzerland (333), the United Kingdom (3,407), and the United States of America (2,093). Sociodemographic characteristics of each country sample, unadjusted results, and details of data cleaning are provided in the Supplemental Material and Methods.

### Willingness to Donate to Different Groups

**Figure 1:**
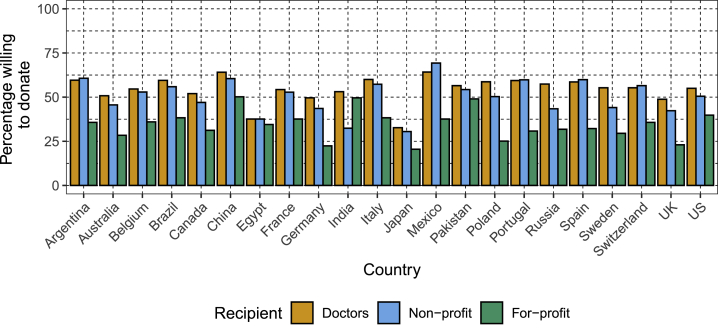
Willingness to Donate Anonymous DNA and Medical Information to Different Recipient Groups, Stratified by Country Each bar shows the percentage of the sample for each country reporting willingness to donate to a particular recipient. The colors of the bars indicate the type of recipient—medical doctors, non-profit researchers, or for-profit researchers.

The majority of participants in the aggregate were either unwilling or unsure about donating their anonymous DNA and medical information for use by researchers (Figure 1, Table S2, Figures S3 and S4).

In general, participants were most willing to donate when the recipient was specified as a medical doctor and least willing to donate when the recipient was a for-profit researcher, but there were exceptions to this pattern (Figure 1, Table S2**,** Figures S1–S4). For example, participants in Poland, Portugal, and Germany were considerably less willing to donate to for-profit users than doctors, whereas the difference was smallest in Egypt, India, and Pakistan.

Within the survey glossary, we explained “anonymous” in more detail as follows: “removal of personal information such as name and date of birth. It is questionable as to whether DNA information can ever be truly anonymous as our DNA code is unique to us and thus, in itself, could be used to identify us. However, in the circumstances we are exploring here, by making DNA and medical information ‘anonymous,’ we mean detaching personal identifiers from it.” What we actually mean here is “de-identified,” but within the pilot work for the survey, we discovered that public participants did not naturally understand this term and “anonymous” was more easily understood. Thus, we added the glossary definition within the survey itself to explain this in more detail.

### Genetics Familiarity

**Figure 2:**
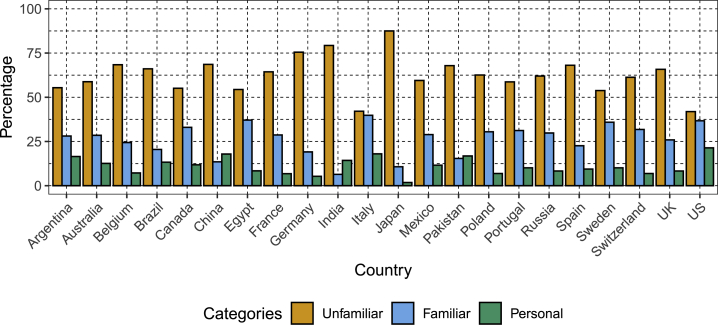
Familiarity with Genetics (Including Familiarity Gained through Personal Experience), Stratified by Country Each bar shows the percentage of the sample for each country reporting level of familiarity with genetics/genomics. Each bar color represents a different self-reported level of familiarity—unfamiliar, conceptual familiarity, or familiarity through personal experience (e.g. through being a patient with a genetic condition).

Familiarity with the concepts of DNA, genetics, and genomics (summarized as “familiarity with genetics”) varies by country (Figure 2), however, the majority of participants in most countries say that they are unfamiliar (total sample: 64.2%; Table S2), and only 35.8% of the total sample say that they have some familiarity with the concepts, including having personal experience of genetics (either through being a patient with a genetic condition, having a family history of a genetic condition, or working with such patients).

### Genetic Exceptionalism

**Figure 3:**
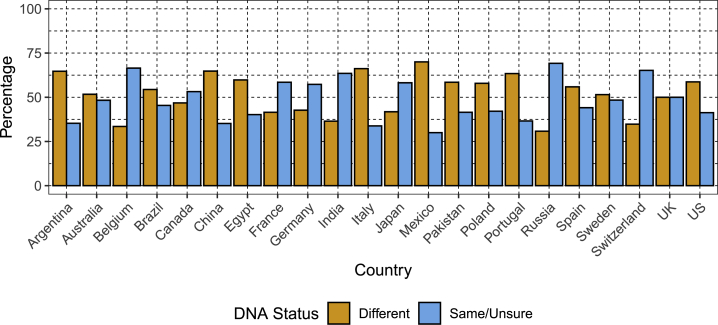
Perception of DNA as Being Different from Other Medical Information (Genetic Exceptionalism) versus DNA as Being the Same (or Unsure), Stratified by Country Each bar shows the percentage of the sample who reported viewing DNA as different to other types of medical information or the percentage who viewed it as being the same or were unsure.

Overall, 53% of the sample viewed DNA as being different from other types of medical information, but views on this differed substantially between countries. For example, over 65% of participants in Mexico and Italy viewed DNA as being different from other types of medical data, whereas only 31% of those in Russia did (Figure 3, Table S2).

### Trust

**Figure 4:**
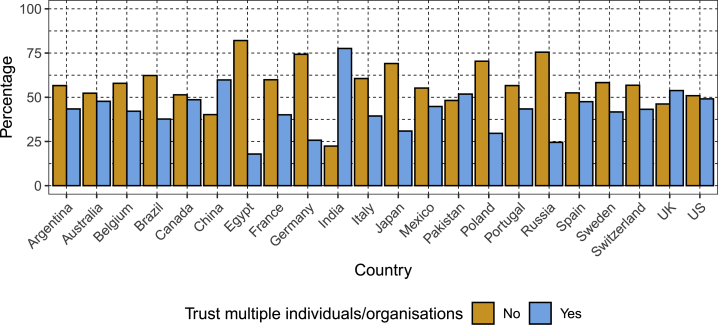
Trust in Donating DNA and Medical Information to More than One User (Including Doctor, Researcher, Company, Government, etc.), Stratified by Country Each bar shows the percentage of the sample who were or were not willing to donate their DNA and medical information to more than one recipient (e.g. medical doctors and for-profit researchers).

Less than half the overall sample reported trusting multiple users (doctors, non-profit researchers, commercial researchers, governments, etc.) with their DNA or health information (Table S2). However, there was substantial variation between countries in terms of trust: more than 50% of participants in China, India, the United Kingdom, and Pakistan trusted more than one actor, compared to fewer than 30% of participants in Egypt, Russia, Germany, and Poland (Figure 4).

### Meta-Analyses

#### Familiarity with Genetics and Willingness to Donate

**Figure 5:**
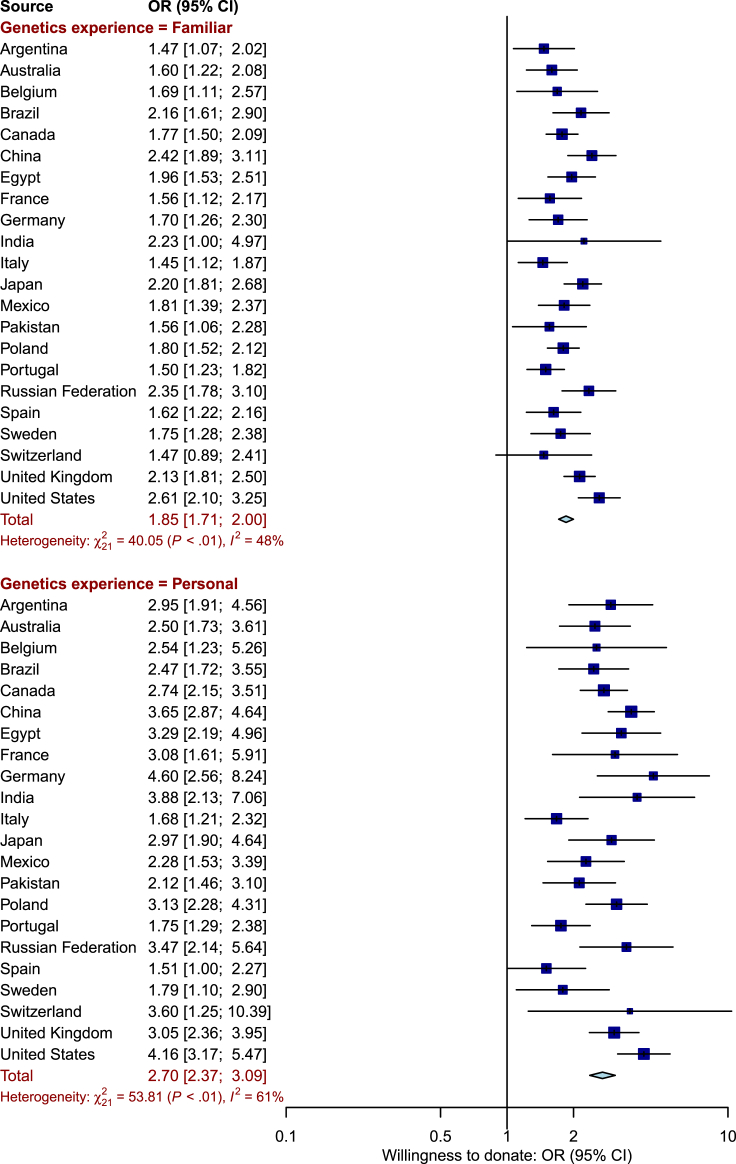
Forest Plot Displaying Association between Willingness to Donate DNA and Medical Information and Familiarity with Genomics, Stratified by Level of Familiarity For each level of familiarity, the forest plot displays the odds ratio (OR) estimate and associated 95% confidence interval (CI) for the sample from each country as a dark blue box with a horizontal line and the overall OR and 95% CI across all countries as a light blue diamond. For each level of familiarity, the comparator is the “unfamiliar” category.

We found that familiarity with the concepts of DNA, genetics, and genomics (termed “familiarity with genetics”) is associated with willingness to donate DNA and medical information. The association, adjusted for sociodemographic factors, is shown in Figure 5. There was evidence for between-country heterogeneity (I^2^ = 48% and I^2^ = 61% for familiarity with genetics and personal experience, respectively). Compared to participants who were unfamiliar with genetics, those who were familiar or had personal experience had greater odds of being willing to donate (odds ratio [OR] = 1.85, 95% CI = 1.11–2.00; OR = 2.70, 95% CI = 2.37–3.09, respectively). The overall pattern of results was similar when considering single groups of recipients of donated DNA and medical information (doctors, non-profit researchers, for-profit researchers); shown in Figures S5–S7.

#### Genetic Exceptionalism and Willingness to Donate

**Figure 6:**
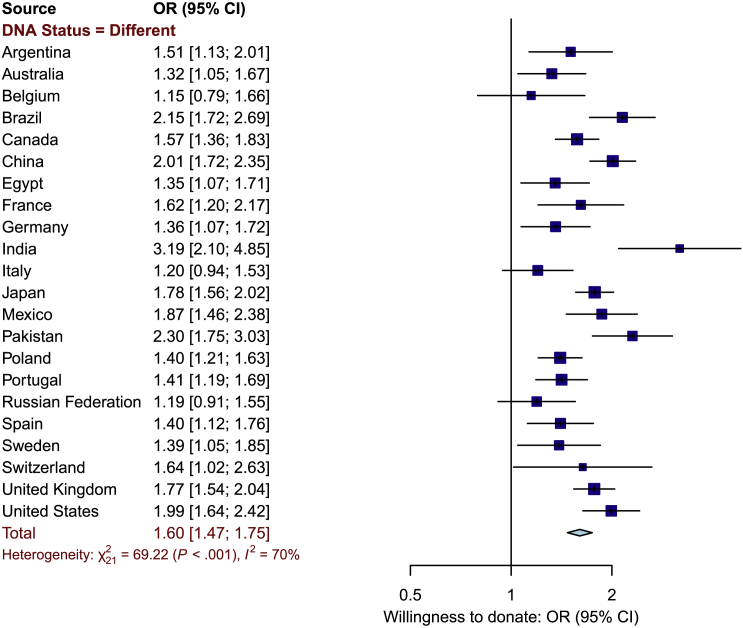
Forest Plot Displaying Association between Willingness to Donate DNA and Medical Information and Perception of DNA as Being Different from Other Medical Data (Genetic Exceptionalism) The forest plot displays the odds ratio (OR) estimate and associated 95% confidence interval (CI) for the sample from each country as a dark blue box with a horizontal line and the overall OR and 95% CI across all countries as a light blue diamond.

We did not find associations between genetic exceptionalism and *decreased* levels of willingness to donate DNA and medical information in any country; there was either no association or a positive association (Figure 6). As expected, there was evidence for heterogeneity (I^2^ = 70%). The pooled OR for the association between genetic exceptionalism and willingness to donate was 1.60 (95% CI = 1.47–1.75).

#### Trust and Willingness to Donate

**Figure 7:**
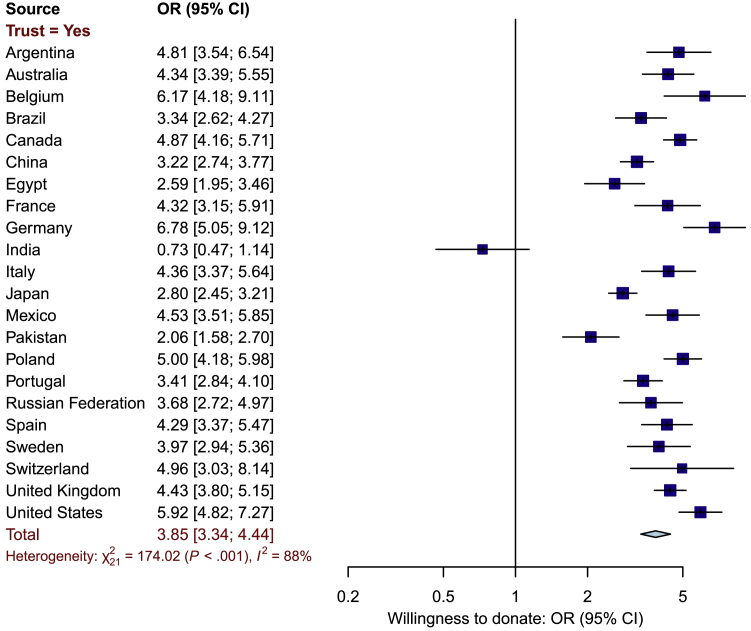
Forest Plot Displaying Association between Willingness to Donate DNA and Medical Information and Trust in Donating DNA and Medical Information to More than One User The forest plot displays the odds ratio (OR) estimate and associated 95% confidence interval (CI) for the sample from each country as a dark blue box with a horizontal line and the overall OR and 95% CI across all countries as a light blue diamond.

In all the samples, apart from India, there was a strong association between trust in multiple actors and willingness to donate DNA and medical information (Figure 7). Although there was substantial heterogeneity (I^2^ = 88%), with the exception of India, this variation was in the *strength* of the positive association between trust and donation rather than the direction of the association. The pooled OR was 3.85 (95% CI = 3.34–4.44).

## Discussion

The “Your DNA, Your Say” project is a very large social sciences study conducted on global public attitudes toward genomic data sharing; it involves a whole mixture of countries with different health systems, population characteristics, and variation in the availability of genomic technology and research. The results show patterns of both consistency and diversity across the globe. What is striking is that public willingness to donate data for research is low across the world; the inclination to trust multiple users with shared data is also low. The reasons for this are complex and multifaceted.

Genomic research, by its very nature, relies on the ability to share data between geographical boundaries because no one single project will unravel the contribution DNA makes to understanding, predicting, and ultimately treating disease in ethnically diverse populations. Our findings highlight how important it is to better focus on familiarizing public audiences with the purposes of genomic research and the reliance of data sharing as part of this. The field of genomics research depends on members of the public’s being comfortable for data about them to be used in research. Although this industry must have exemplary models of conduct that make them worthy of public trust, being able to authentically communicate this is likely to help increase the willingness of publics to donate their data. We will explore what publics believe will help increase the trustworthiness of researchers in future papers.

Attitudes toward donation are shaped by with whom data are shared and by whom they are most likely to be used. Across our sample, people are less willing to donate data to for-profit users than they are to non-profit organizations or doctors. This echoes prior work in the UK, the USA, Australia, Egypt, China, and across Europe.^7^, ^8^, ^9^, ^10^^,^^13^ In some countries, notably India but to a lesser extent the USA, China, and Pakistan, the distinction between non-profit and for-profit research is less clear-cut. This may reflect the differing local roles of the private sector in healthcare and research. In India, for example, studies suggest a preference for private-sector rather than public healthcare, whereas healthcare in the USA is predominantly delivered in the private sector.^24^ The between-country differences indicate that nuanced approaches, tailor-made to each cultural setting, are required when explaining data sharing processes.

The variable responses to willingness to donate to multiple users, a situation that most accurately reflects current research practice, suggests that there is a continuing need for clearer messages about the collaborative nature of contemporary genomics research (including the flow of data that may be accessed by multiple entities multiple times and for different uses) and the role of partnership between non-profit and for-profit sectors.^25^ It also suggests the need to explore factors that deter people from donating to for-profit researchers. The overall analysis further identifies countries, notably Germany and Japan, where participants were less willing to donate their DNA and health data overall—findings which echo previous work on concerns around the use of genetic data.^26^

We highlight the important role of trust in shaping people’s willingness to donate DNA and medical data. In our analysis, trust is consistently associated with the willingness to donate, albeit with varying strength. This finding corresponds with our own previous work and prior national and comparative studies, where available, that provide evidence for the importance of building trust through both clarity of purpose and demonstrably trustworthy technical infrastructures and governance arrangements.^10^^,^^12^^,^^13^^,^^16^ However, the varying relationship between trust and willingness to donate suggests that trust in data users may not mean the same thing everywhere.^27^ Further, our findings relate to trust in actors in people’s own countries, but attitudes to data users outside one’s own country may be less supportive; this has implications for the local viability of international research initiatives.^26^^,^^28^ This suggests the importance of fostering trust locally through governance regimes that are sensitive to public expectations and concerns.

We found that those people who are most familiar with genetics, and particularly those with personal experience through being a patient, having a family history of an inherited condition, or working in the genetics field, are consistently those most willing to donate their DNA and health data. Conversely, lack of familiarity with genetics was associated with reticence about data donation. Thus, the field of genomics needs to do much more to explain and invite public debate on the global contribution genomic research makes toward understanding, predicting, and ultimately treating disease (i.e., increasing familiarity with the *purpose of* genetic research). This message extends work that has shown that prior engagement is a key factor in shaping willingness to participate in genetics research.^13^^,^^26^ We also found that there is consistently low familiarity with the concepts of DNA, genetics, and particularly genomics around the world. In only Italy and the USA do more people claim to be familiar than unfamiliar. Previous research has similarly found that, in the UK for example, only 34% had some knowledge of genomics.^29^ In some cases, however, the level of familiarity we describe is somewhat lower than has been identified in previous research—particularly in Japan, possibly because the term “genome” in Japanese is an English loanword.^30^

The low level of familiarity suggests a need to recognize challenges associated with communicating genetic information. However, we should be cautious about equating familiarity with knowledge and are fully aware that familiarity is not a proxy measurement for understanding. Among our authorship are experienced genetic counsellors who are cognizant of the importance of helping patients to “make meaning” of the concepts of genomics, for example, by explaining why genetics is relevant to us in society and what the technology can offer us, as a priority over explaining the technical scientific concepts, such as DNA’s consisting of four chemical bases (“knowledge, literacy, understanding”). This is because we know empirically that publics understand genomics through personal and family experience in terms that are not necessarily those of a technical scientific vocabulary.^27^ Our findings suggest the continued need to consider how awareness of genomics (as opposed to literacy or scientific knowledge) can be useful in making decisions about genomic data donation.

Interestingly, diverse responses were found for the question of genetic exceptionalism. Whether DNA is similar to or different from other medical information is a repeated and contested question in discussions of ethics and law related to genetics.^14^^,^^15^ In line with our previous work, the findings of the current study suggest that perceiving genetic material as exceptional does not reduce the willingness to donate, and indeed in many cases, it increases the odds of donation.^31^ Therefore, in line with our recommendations to increase familiarity with genetics, a useful place to start (that may indeed increase willingness to engage in research) could be to highlight and encourage public debate about the differences and similarities between genomic and other medical data.

### Limitations of Study

The limitations of the study and design have been published separately.^17^ As an exploratory cross-sectional online survey, the study is limited in that it captures intended behavior at a single time point. This may not translate to practice, and there is some evidence that people are more hesitant about hypothetical genetic research than they are in reality.^32^^,^^33^

Two sources of potential variability should be noted. Participants in India and Pakistan completed the survey differently from other sites, either at a central location or on a tablet provided by field researchers. This may have introduced variation in the responses. However, this variability is outweighed by the value of obtaining responses from lower resource contexts, which are often excluded from research into public attitudes toward genomics. Second, although close attention was paid to creating accurate translations, translating terms such as genomics, data, or pseudonymization relies on the available terms in each language and the cultural meanings these terms carry. This may introduce noise in the data analysis and requires further investigation. Every translated survey was also back translated into English; the process of translation and back-translation involved experts with knowledge of genomics and data sharing so that decisions could be made about the translations in relation to construct and content validity. However, other than an expert view on this, because this is a hypothesis-generating study, no further validation was possible.

Our results are valuable for tentative conclusions and hypotheses but do not indicate views of all people from each of the countries studied.

### Conclusions

The “Your DNA, Your Say” project is a large social sciences study that provides empirical analysis of the global variation in public perspectives about genomic data. The results demonstrate the importance of familiarity and trust in the collection and sharing of genomic and health data.

We conclude that there are clear messages for policy makers, genomic researchers, clinicians, and governments who are implementing genomic research strategies that use data that cross geographical boundaries. More needs to be done to familiarize public audiences with (and create two-way dialogue around) the following: the purpose of global genomic research and why data donation and subsequent sharing is integral to this; why a partnership between doctors, non-profit, and for-profit industries is necessary; and what the relevance of genomic technology is to our lives. To support this latter point, we advocate increasing familiarity with the implications of genome research and the applications of technology (as opposed to prioritizing genomic literacy or knowledge) by using the similarities and differences between genomic and other health data as a springboard for these conversations.

Maximizing societal benefits from genomic data involves acknowledging and responding to the factors that shape the decision to donate DNA across social, cultural, and legal contexts. Public benefit and the protection of public interests can only be delivered if clear, transparent, and authentic information is provided to enable publics to consider if and why their contribution to a partnership with researchers is important. The global research community not only needs to be worthy of trust by the public, but also urgent steps need to be taken to communicate this.

## Declaration of Interests

The authors declare no competing interests.

## Web Resources

Global Alliance for Genomics and Health, https://www.ga4gh.org/
Your DNA, Your Say Overview, https://societyandethicsresearch.wellcomegenomecampus.org/project/your-dna-your-say
Your DNA, Your Say Survey, https://www.yourdnayoursay.org/

## Data and Code Availability

The full dataset of survey responses has not yet been deposited in a public repository because additional countries are joining the research and we are waiting to finish recruitment. Once recruitment is completely finished, the full dataset will be published at https://www.wgc.org.uk/ethics and publicly available without restriction for anyone to access, download, and analyze.

## Supplemental Data

Document S1. Figures S1–S7, Table S3, and Supplemental Material and Methods

Table S1 Sociodemographic Characteristics of Participants from Each Country

Table S2 Unadjusted Results for Full Dataset, Stratified by Country

Document S2. Article plus Supplemental Information
