# Supplementary material for: Global Public Perceptions of Genomic Data Sharing: What Shapes the Willingness to Donate DNA and Health Data?
Source: Am J Hum Genet. 2020 Sep 17;107(4):743–52. doi: 10.1016/j.ajhg.2020.08.023 (PMC7536612; doi:10.1016/j.ajhg.2020.08.023)
Supplement: Document S1. Figures S1–S7, Table S3, and Supplemental Material and Methods [file mmc1.pdf]

The American Journal of Human Genetics, Volume *107*

Supplemental Data

Global Public Perceptions of Genomic Data Sharing: What Shapes the Willingness to Donate DNA and Health Data?

Anna Middleton, Richard Milne, Mohamed A. Almarri, Shamim Anwer, Jerome Atutornu, Elena E. Baranova, Paul Bevan, Maria Cerezo, Yali Cong, Christine Critchley, Josepine Fernow, Peter Goodhand, Qurratulain Hasan, Aiko Hibino, Gry Houeland, Heidi C. Howard, S. Zakir Hussain, Charlotta Ingvoldstad Malmgren, Vera L. Izhevskaya, Aleksandra Jędrzejak, Cao Jinhong, Megumi Kimura, Erika Kleiderman, Brandi Leach, Keying Liu, Deborah Mascalzoni, Álvaro Mendes, Jusaku Minari, Nan Wang, Dianne Nicol, Emilia Niemiec, Christine Patch, Jack Pollard, Barbara Prainsack, Marie Rivière, Lauren Robarts, Jonathan Roberts, Virginia Romano, Haytham A. Sheerah, James Smith, Alexandra Soulier, Claire Steed, Vigdís Stefànsdóttir, Cornelia Tandre, Adrian Thorogood, Torsten H. Voigt, Anne V. West, Go Yoshizawa, and Katherine I. Morley

Supplementary Material

Figure S1 Willingness to donate anonymous DNA and medical information to at least two recipient groups, by country

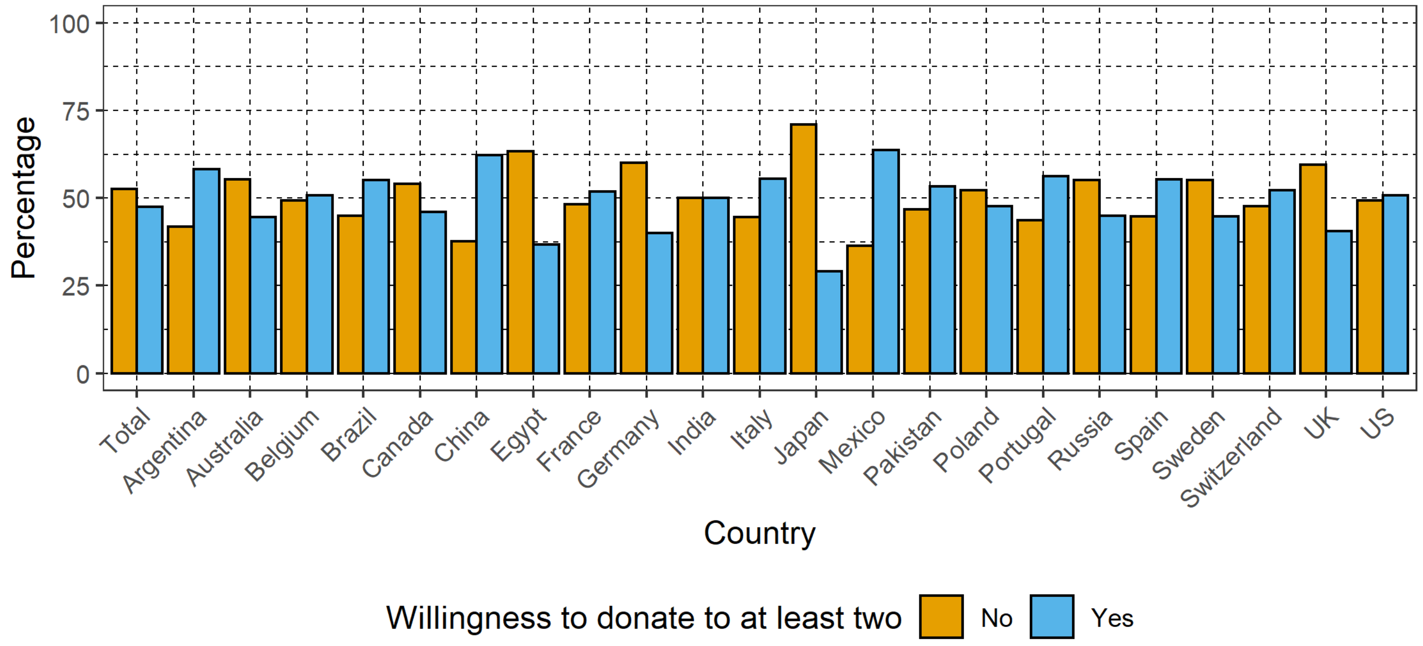

Figure S2: Willingness to donate anonymous DNA and medical information to doctors

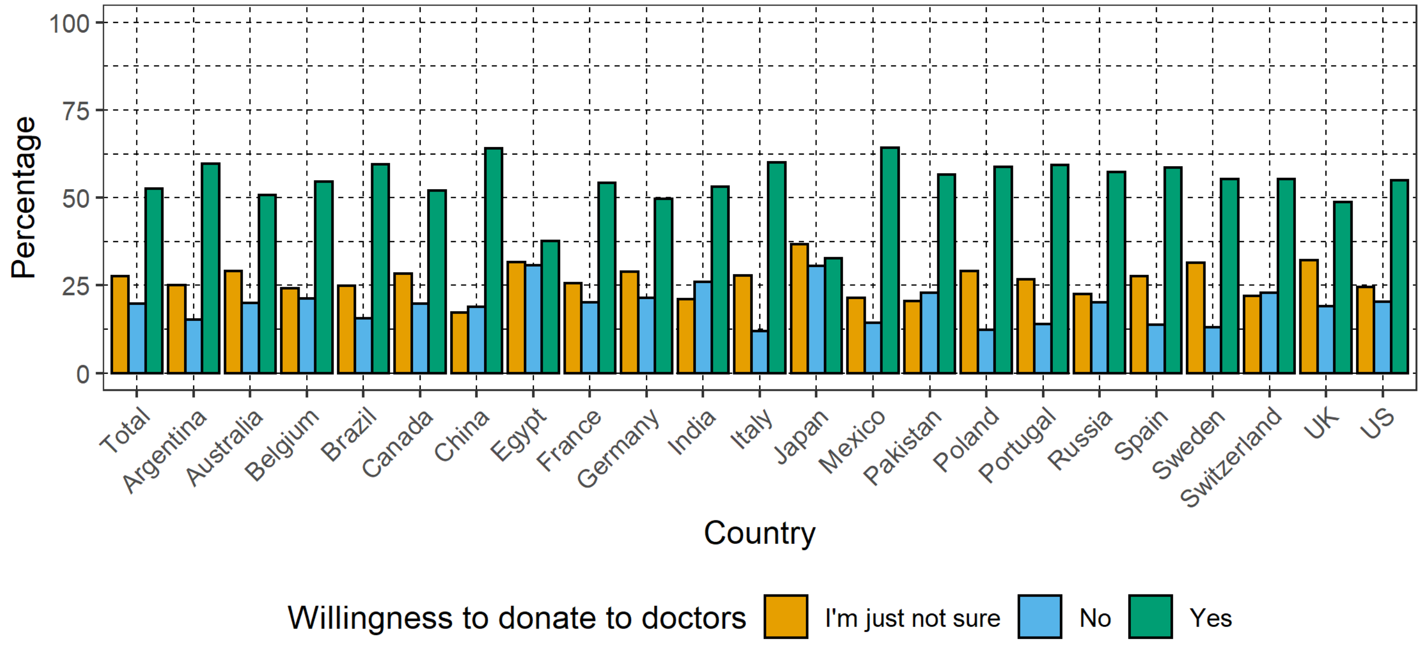

Figure S3 Willingness to donate anonymous DNA and medical information to non-profit researchers

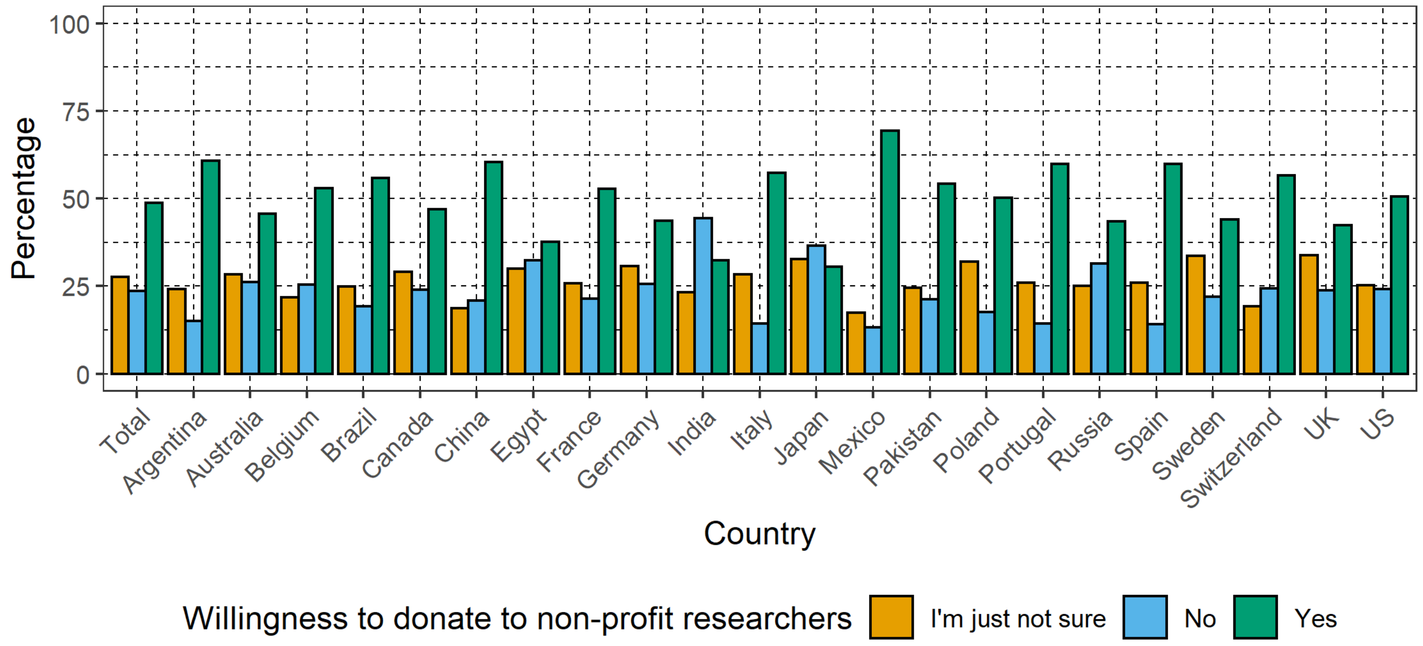

Figure S4: Willingness to donate anonymous DNA and medical information to for-profit researchers

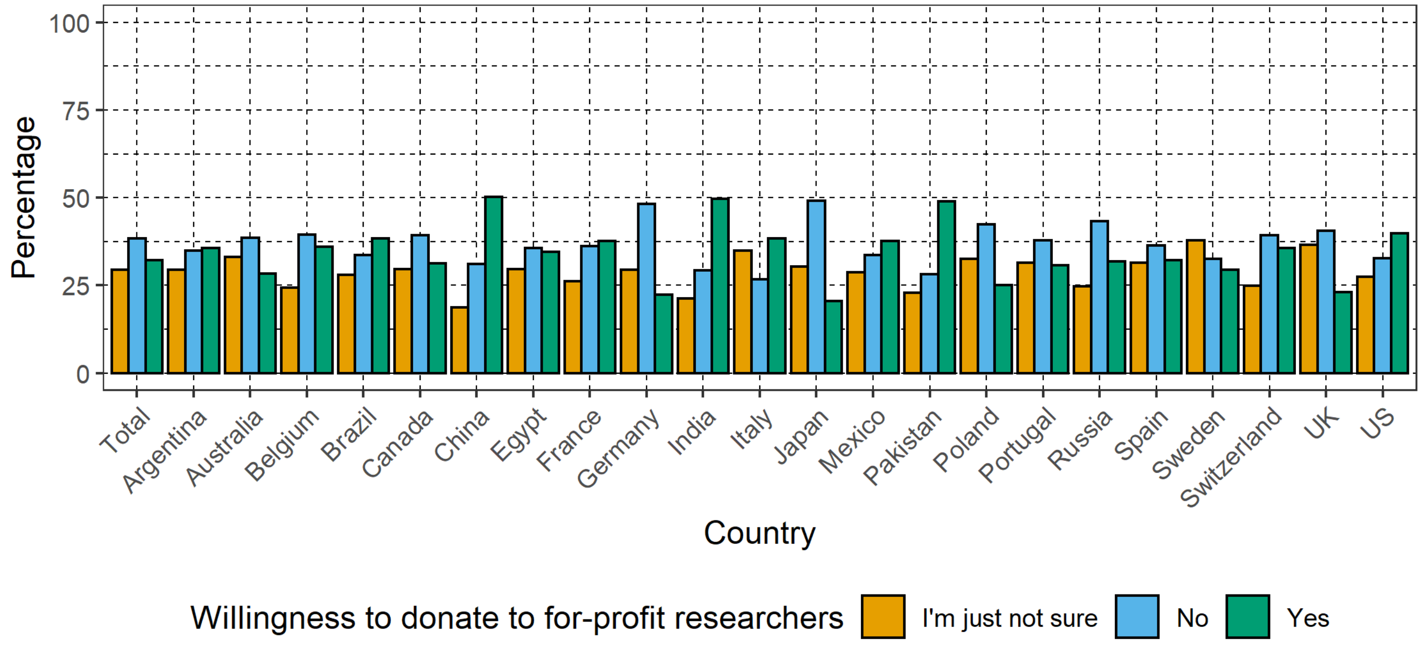

Figure S5: Associations between familiarity with genetics (including familiarity gained through personal experience) and willingness to donate for use by medical doctors

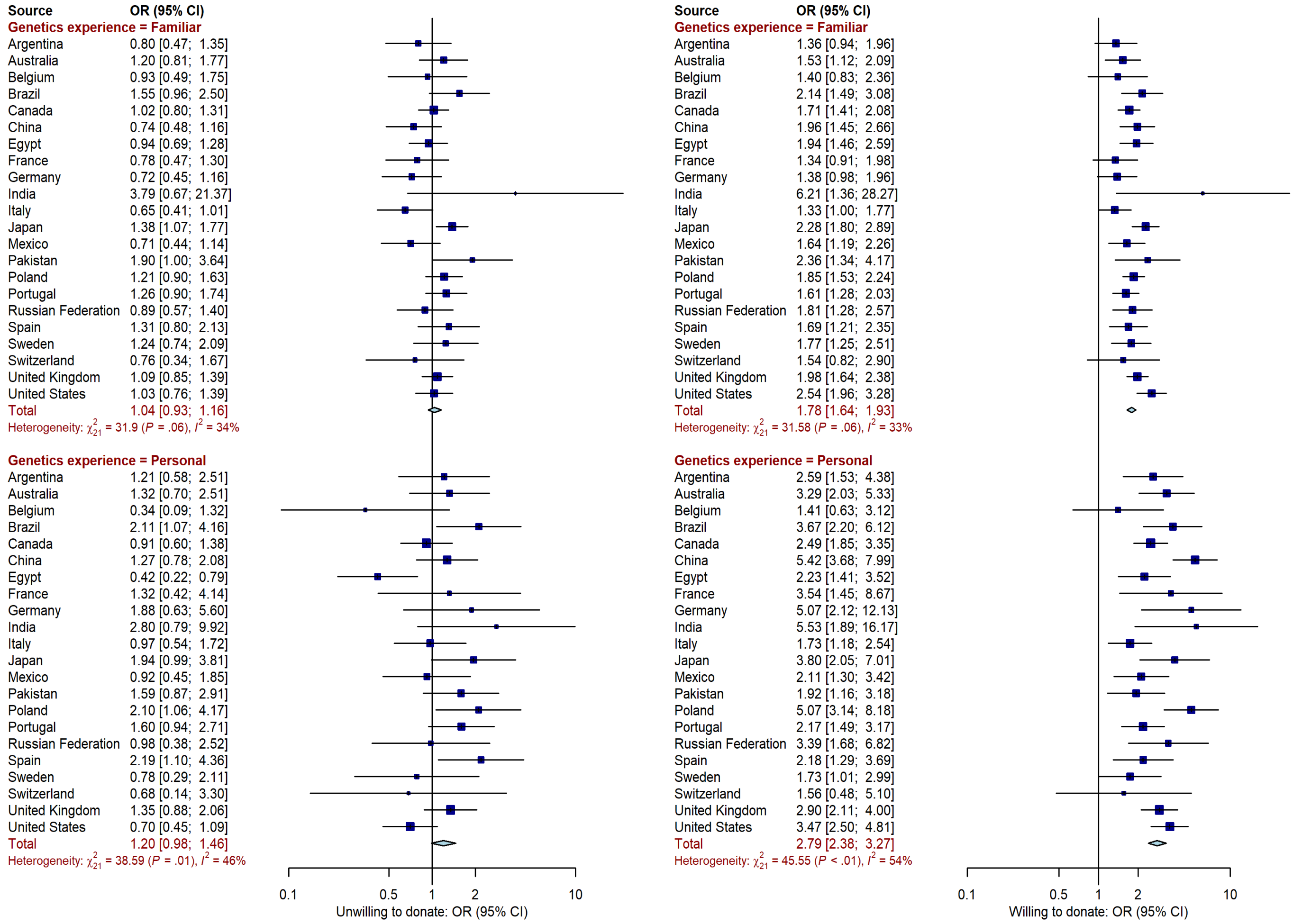

Figure S6: Associations between familiarity with genetics (including familiarity gained through personal experience) and willingness to donate for use by non-profit researchers

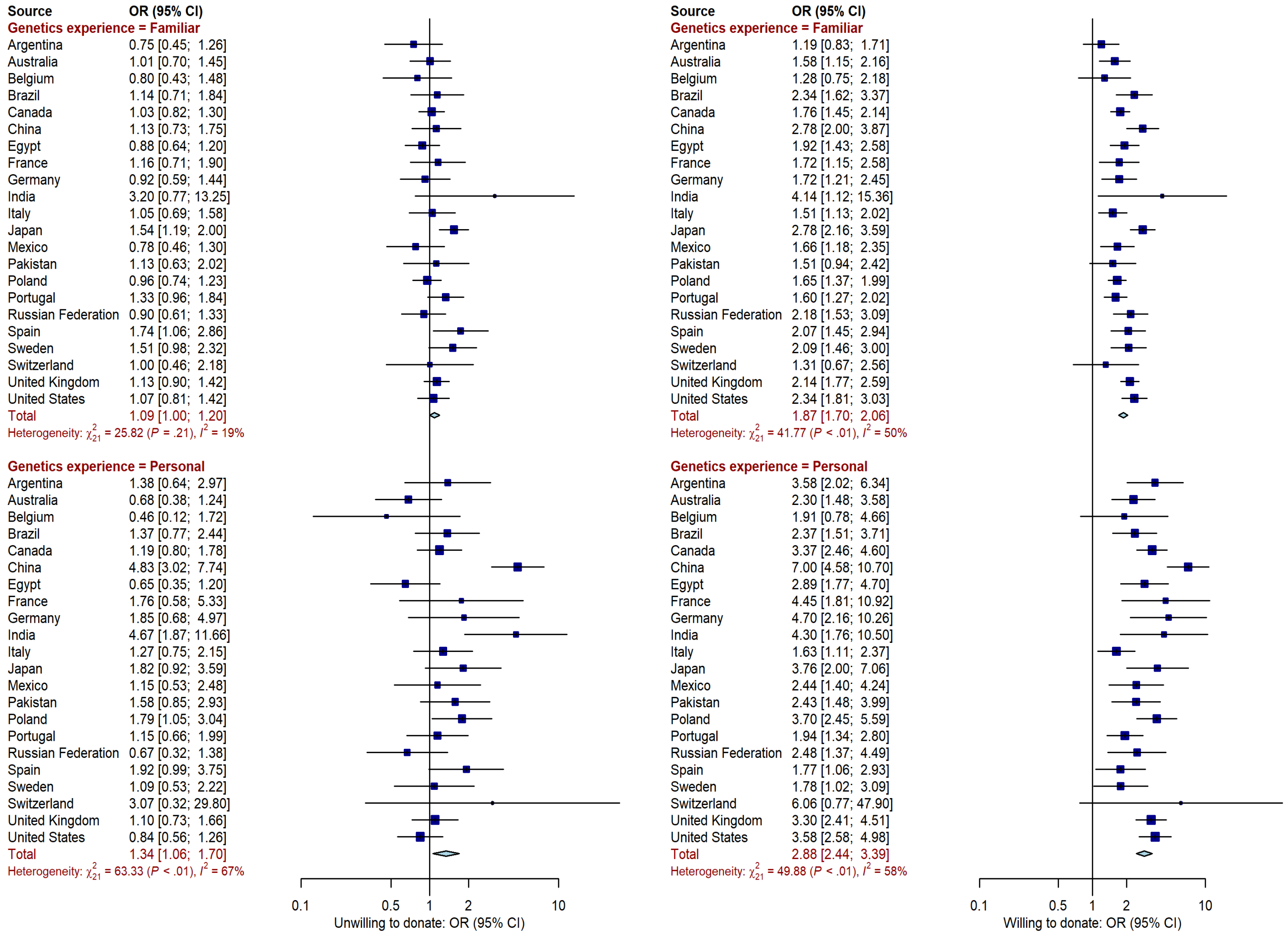

Figure S7: Associations between familiarity with genetics (including familiarity gained through personal experience) and willingness to donate for use by for-profit researchers

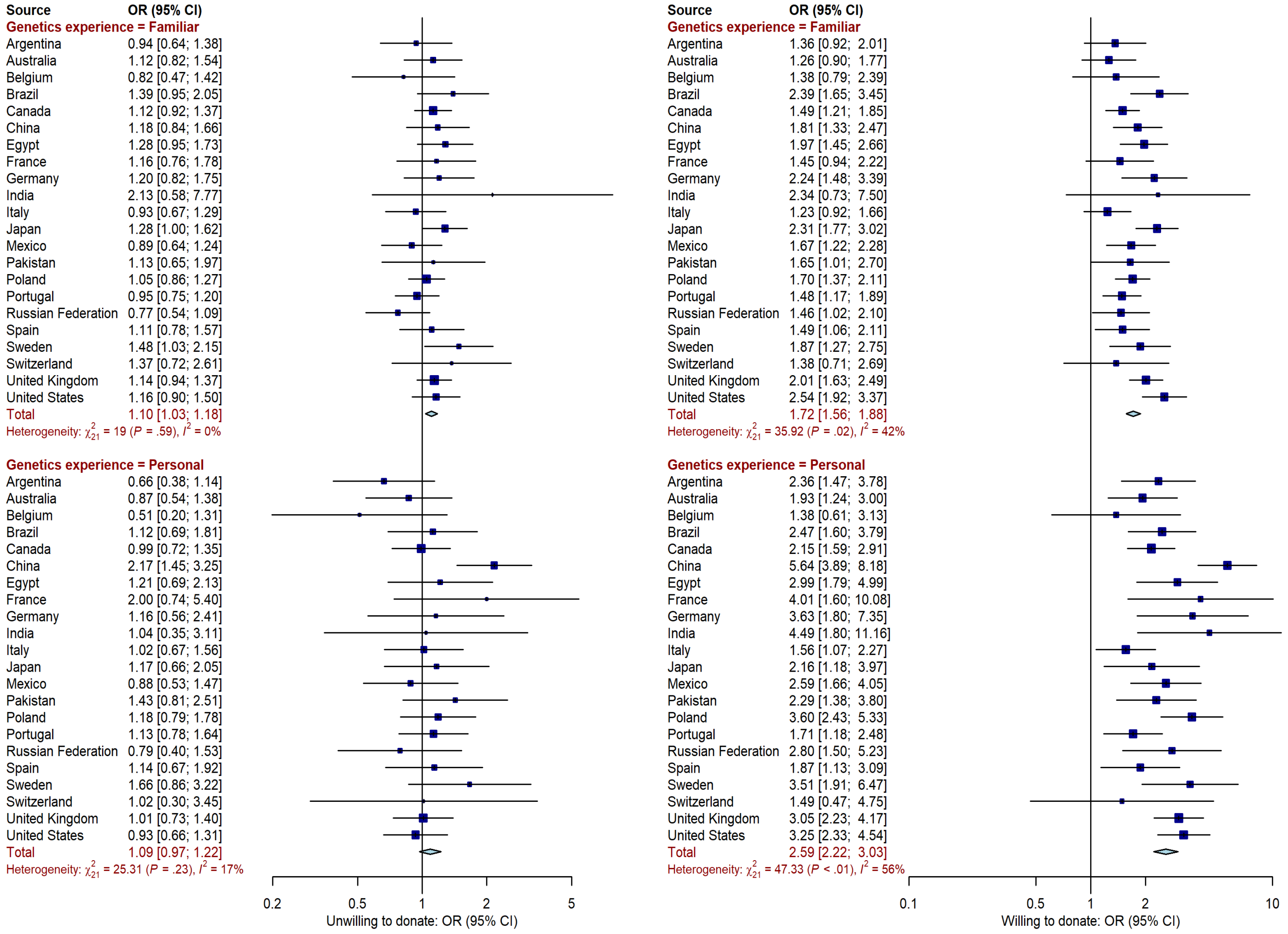

Table S1: Sociodemographic characteristics of country samples. See accompanying Excel file labelled Table S1

Table S2: Descriptive analyses of variables across the countries sampled. See accompanying Excel file labelled Table S2

Table S3: Predictor-outcome associations

Predictor	Outcome
Genetics familiarity	Willingness to donate (multiple recipients)
	Willingness to donate to medical doctors
	Willingness to donate to non-profit researchers
	Willingness to donate to for-profit researchers
Genetic exceptionalism	Willingness to donate (multiple recipients)
Trust at least two types of individuals/organisations receiving the data	Willingness to donate (multiple recipients)

Supplemental Methods:

Donating DNA and medical information

Participants were asked whether they would donate "anonymous"* DNA and medical information for use in research by (a) medical doctors; (b) non-profit researchers; (c) for-

* Here we mean 'de-identified', but pilot work showed that 'anonymous' was more easily understood by participants. A glossary definition within the survey explained 'anonymous' as: 'removal of personal information such as name and date of birth. It is questionable as to whether DNA information can ever be truly anonymous as our DNA code is unique to us and

profit researchers. Participants could answer ‘Yes’, ‘No’, or ‘Unsure’. Three measures of willingness retained the three response options:

- Donate to doctors: donate to medical doctors
- Donate to non-profit: donate to non-profit researchers (e.g. from universities);
- Donate to for-profit: donate to for-profit researchers (e.g. from pharmaceutical companies).

We created a fourth variable that combined the above and had the options ‘Yes’ or ‘No’:

- Donate to multiple recipients: willingness to donate at least two of: doctor, non-profit researcher; for-profit researcher.

Familiarity with genetics

Familiarity was derived from two questions. The first was “Are you familiar with DNA, genetics, or genomics?". If a respondent answered in the affirmative, they could specify by checking one or more of the options below and were classified as ‘familiar with genetics’. If participants checked one or more of the options in bold, they were classified as having ‘personal experience’ of genetics. We defined ‘personal experience’ as having experienced the personal significance of serious, inherited disease (e.g. a genetics ‘patient’ or professional who works with or had exposure to the impact of genetic disease). Participants without this experience were categorised as “Familiar" or “Unfamiliar" based on their response to the first question.

- Person interested in ancestry/genealogy websites
- Direct-to-consumer company customer (e.g. 23andMe)
- Biobank participant

---

thus, in itself, could be used to identify us. However, in the circumstances we are exploring here, by making DNA and medical information 'anonymous', we mean detaching personal identifiers from it.’

- **Person with a genetic condition or family history of an inherited condition**
- **Genetic health professional (clinical geneticist, genetic counsellor, clinical lab staff, work in genetics services in a clinical setting)**
- **Genetic researcher/student (e.g. lab, bioinformatician, management, social science, ethics, policy, public health, public engagement, administration in a genetics institute, non-profit or for-profit)**
- Non-genetics researcher/student (e.g. management, social science, ethics, policy, public health, public engagement, administration)
- Non-genetics health professional (e.g. nurses, GPs, surgeons, hospital specialists, hospital administration staff, medical students)
- Research participant in any genetics research (e.g. as a healthy volunteer or as a person with a particular genetic condition or family history of an inherited condition)
- Other, please specify

We have published an explanation of why 'personal experience of genetics' is relevant to shaping attitudes in Middleton et al (2020).

Genetic exceptionalism

Participants' perception of DNA information was collected via the question "Is DNA information different to medical information – what do you think?". Response options were "Different", "The same", "I'm not sure". The latter two categories were collapsed for analysis.

The rationale for collapsing the data was because we wanted to focus on participants who had clear (as opposed to unsure) views on this. More details about the relevance of genetic exceptionalism can be found in the above Middleton et al (2020) paper.

Sociodemographics

Sociodemographics are shown in Table S1. Age was collected in ten-year categories from 16 onwards. Due to fewer responses in younger and older categories these were collapsed into categories of "30 years and under", "31–40","41–50", "51–60", and "61 years and older" for analysis. Gender was self-described "Female" or "Male". Whether participants had children was determined by a "Yes" or "No" answer. Level of education was categorised as "Tertiary", "Secondary", "Primary" or "Other" based on structured responses and free-text descriptions,

standardised across the countries sampled. This was collapsed to a binary indicator of tertiary education for multivariable analyses. Religiosity was determined by the question “Independent of whether you attend religious services or not, would you say you are … ?" with options “A religious person" or “Not a religious person".

Data cleaning

Only completed surveys were included. To remove responses where individuals had not engaged with the content, we only included surveys in which all mandatory questions were completed and which took more than five minutes to complete. Piloting showed that it was not possible to engage with all the survey questions and complete them in such a short period of time. Participants had the ability to start the survey, pause and come back to it another time and so some participants chose to complete the survey over several days. For those who completed the survey in one sitting, the average time it took to complete across all the countries was 22 minutes.

As there were multiple questions regarding willingness to donate, we were able to identify and remove data from respondents that were completely inconsistent (e.g. responding to one question that they would not consider donating DNA and medication information under any circumstances, but then responding that they would donate and accept risk of being identified via their donated data in another question). Approximately 5% of participants gave inconsistent responses, this was similar across country subsamples.

The background, context, methods, limitations and English-speaking and German-speaking results have already been peer reviewed and published (1-7).

Supplemental References

1. Middleton A, Milne R, et al (2020) Members of the public in the USA, UK, Canada and Australia expressing genetic exceptionalism say they are more willing to donate genomic data. European Journal of Human Genetics, vol. 28, issue 4, pp 424-434, April
2. Voigt TH, Holtz V, Niemiec E, Howard HC, Middleton A, Prainsack B (2020). Willingness to donate genomic and other medical data: results from Germany. European Journal of Human Genetics. Published online ahead of print, 1st April. Available at: https://doi.org/10.1038/s41431-020-0611-2
3. Milne R, Morley KI, Howard H… Atutornu J, Farley L, Middleton A, et al (2019) Trust in genomic data sharing among members of the general public in the UK, USA, Canada and Australia. Human Genetics, vol. 138, issue 11-12, pp 1237-1246, December
4. Middleton A, Milne R, et al (2019) Attitudes of publics who are unwilling to donate DNA data for research. European Journal of Medical Genetics, vol. 62, issue 5, pp 316–323, May
5. Middleton A, Niemiec E, Prainsack B… Farley L, Morley KI, et al (2018) 'Your DNA, Your Say': global survey gathering attitudes toward genomics: design, delivery and methods. Personalized Medicine, vol. 15, issue 4, pp 311-318, July
6. Middleton A (2018) Society and personal genome data. Human Molecular Genetics, vol. 27, issue R1, pp R8–R13, March
7. Middleton A (2017). Data Sharing and Participation: Your DNA, Your Say. The New Bioethics, vol. 23, issue 1, pp 74-80
